# Artificial Self-Sufficient P450 in Reversed Micelles

**DOI:** 10.3390/molecules15052935

**Published:** 2010-04-27

**Authors:** Hidehiko Hirakawa, Noriho Kamiya, Yutaka Kawarabayasi, Teruyuki Nagamune

**Affiliations:** 1 Department of Bioengineering, School of Engineering, The University of Tokyo, 7-3-1 Hongo, Bunkyo-ku, Tokyo 113-8656, Japan; E-Mail: hirakawa@bio.t.u-tokyo.ac.jp (H.H.); 2 Center for NanoBio Integration, The University of Tokyo, 7-3-1 Hongo, Bunkyo-ku, Tokyo 113-8656, Japan; 3 Department of Chemistry and Biotechnology, School of Engineering, The University of Tokyo, 7-3-1 Hongo, Bunkyo-ku, Tokyo 113-8656, Japan; 4 Department of Applied Chemistry, Graduate School of Engineering & Center for Future Chemistry, Kyushu University, Motooka 744, Fukuoka 819-0395, Japan; E-Mail: nori_kamiya@mail.cstm.kyushu-u.ac.jp (N.K.); 5 New Energy and Industrial Technology Development Organization, 19F Muza Kawasaki Building, 1310, Omiya-cho, Saiwai-ku, Kawasaki City Kanagawa 212-8554, Japan; E-Mail: kawarabayashiytk@nedo.go.jp (Y.K.)

**Keywords:** artificial self-sufficient cytochrome P450, cofactor regeneration, transglutaminase, reversed micelles

## Abstract

Cytochrome P450s are heme-containing monooxygenases that require electron transfer proteins for their catalytic activities. They prefer hydrophobic compounds as substrates and it is, therefore, desirable to perform their reactions in non-aqueous media. Reversed micelles can stably encapsulate proteins in nano-scaled water pools in organic solvents. However, in the reversed micellar system, when multiple proteins are involved in a reaction they can be separated into different micelles and it is then difficult to transfer electrons between proteins. We show here that an artificial self-sufficient cytochrome P450, which is an enzymatically crosslinked fusion protein composed of P450 and electron transfer proteins, showed micelle-size dependent catalytic activity in a reversed micellar system. Furthermore, the presence of thermostable alcohol dehydrogenase promoted the P450-catalyzed reaction due to cofactor regeneration.

## 1. Introduction

Certain enzymes, especially oxidoreductases, prefer hydrophobic compounds as substrates. However, the poor solubility of such compounds in water is a critical problem for industrial applications. To overcome this problem enzymatic reactions in non-conventional media, such as water-immiscible organic solvents [1], two-phase systems of water and organic solvent [2], ionic liquids [3], microemulsions [4] and reversed micelles [5] have been developed. The reversed micellar system, which has nano-meter scaled water pools in an organic solvent, can retain hydrophilic molecules, such as proteins and cofactors in a water pool, while hydrophobic compounds are dissolved in the organic solvent. Because of this characteristic, many reactions of cofactor-dependent enzymes, such as dehydrogenase, have been reported in reversed micellar systems [6,7,8,9,10]. 

Cytochrome P450s (P450s), which are heme-containing monooxygenases, can catalyze a wide variety of stereo- and regioselective oxidations of hydrophobic compounds with molecular oxygen [11]. To convert heme-binding oxygen to active species, it is necessary to accept electrons from NAD(P)H via electron transfer proteins. To date, several electron transfer pathways have been discovered and P450s are classified according to their preferred electron transfer system [12]. Most bacterial P450s belong to class I, which require two separate proteins, ferredoxin, which contains [2Fe-2S], and ferredoxin reductase, which contains FAD. These P450s are good candidates for enzymatic reactions in reversed micelles because they require water-soluble cofactors and they prefer hydrophobic compounds as substrates. Micelles can exchange water pool contents by continuously repeated association, fusion and dissociation [13]. Nonetheless, it is unlikely that proteins in different micelles will interact with each other in appropriate orientations during micelle fusion. Therefore, a single micelle must contain P450 and its electron transfer proteins to complete P450-catalyzed reactions in a reversed micellar system. Reactions by a bacterial P450 and its electron transfer protein in reversed micelles and in microemulsions have been reported [14,15,16]. However, high protein concentrations are required for single micelles to contain every component. 

**Figure 1:**
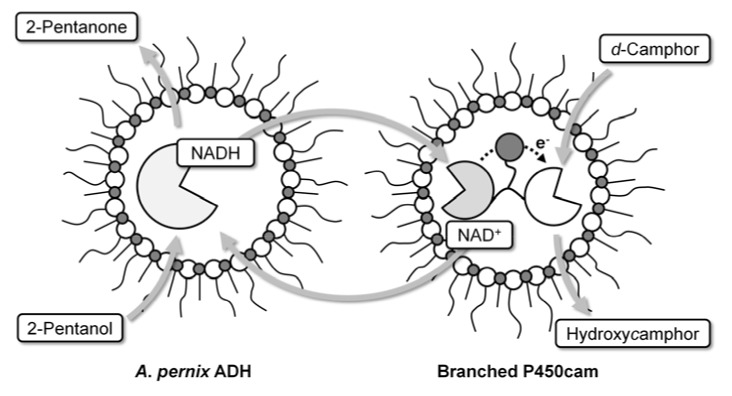
Reaction scheme for *d*-camphor hydroxylation by branched P450cam with cofactor regeneration in a reversed micellar system.

Recently, we have reported a site-specific branched fusion protein (branched P450cam), which is composed of one molecule each of a P450 from *Pseudomonasu putida* (P450cam) and its electron transfer proteins, putidaredoxin (PdX) and putidaredoxin reductase (PdR) [17]. The fusion protein is self-sufficient and shows high catalytic activity due to intramolecular electron transfer. This suggests that the fusion protein should function in reversed micelles, even at catalytic concentration. Here, we report *d*-camphor hydroxylation by branched P450cam in a reversed micellar system. We also report a coupled reaction with regeneration of NADH, which is consumed during the hydroxylation of *d*-camphor, by organic solvent-activated alcohol dehydrogenase from *Aeropyrum pernix* (*A*. *pernix* ADH) (Figure 1).

## 2. Results and Discussion 

Dioctylsulfosuccinate sodium salt (AOT)/isooctane reversed micelles have been widely used for enzymatic reactions because of stable and large micelle formation [18,19]. In this study, we used a CTAB/2-pentanol/isooctane reversed micellar system, which contains 2-pentanol as a cosurfactant, because AOT could not form stable micelles in the presence of 50 mM potassium ions, which are necessary for maximal activity of P450cam [20]. 2-Pentanol is also a substrate of *A*. *pernix* ADH for the regeneration of NADH. 

### 2.1. Hydroxylation of d-camphor by branched P450cam in reversed micelles

**Figure 2:**
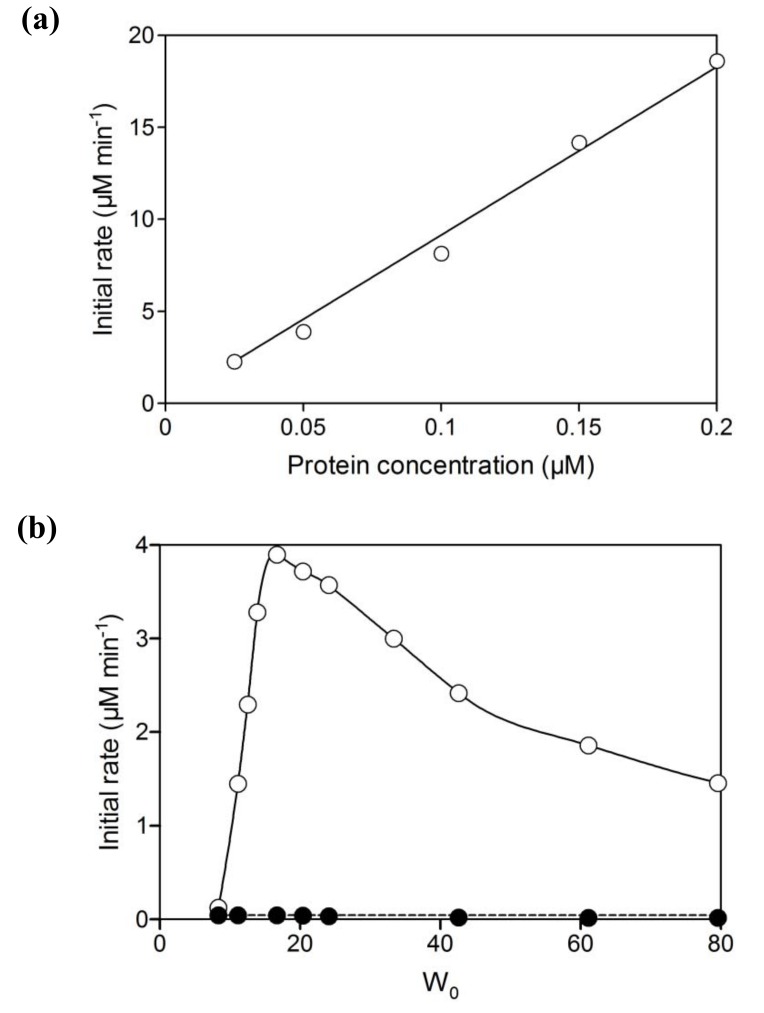
(a) Relationship between the initial rate of NADH oxidation and the concentration of branched P450cam at a water content (W_0_) of 16.7. The reaction mixture contained 5 mM *d*-camphor and 80 μM NADH. (b) Effect of W_0_ on the initial activities of branched P450cam (open circles) and an equimolar mixture of PdR, PdX and P450cam (closed circles). The reaction mixtures contained 0.05 μM protein, 5 mM d-camphor and 80 μM NADH. The reversed micelle solution containing PdR, PdX and P450cam was prepared by adding a mixture of PdR, PdX and P450cam to a starter reversed micelle solution (see experimental section).

**Figure 3:**
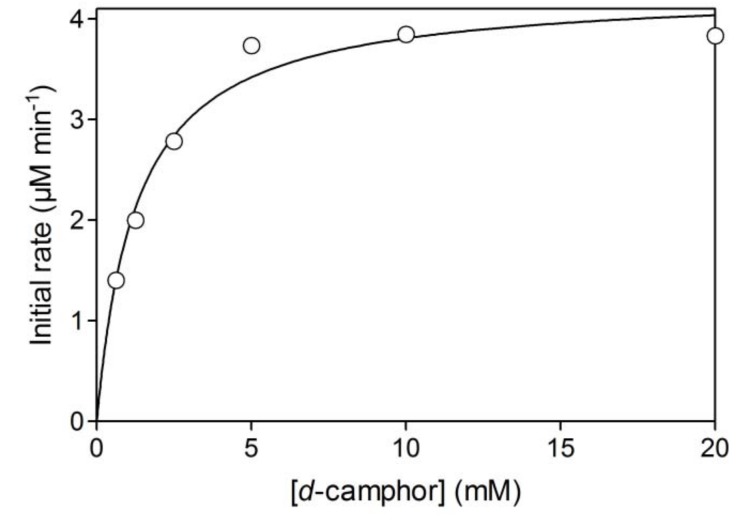
Relationship between initial rate of NADH oxidation and the concentration of *d*-camphor at a W_0_ of 17. The reaction mixture contained 0.05 μM branched P450cam and 80 μM NADH.

**Figure 4:**
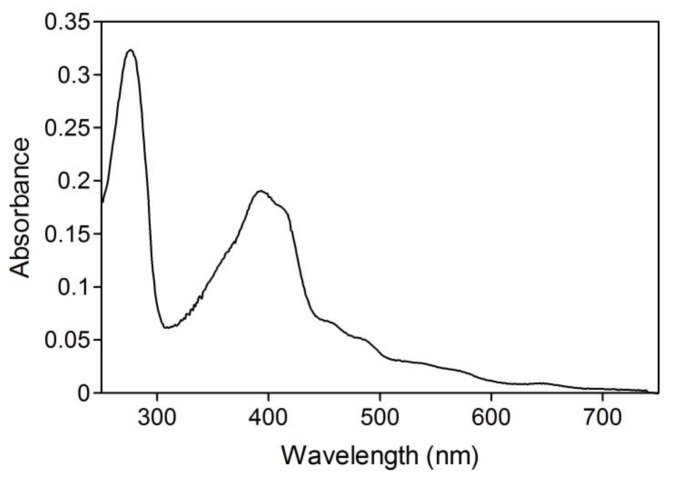
UV-Vis spectrum of 2 μM branched P450cam with 5 mM *d*-camphor in the reversed micellar system at a W_0_ of 17.

Initial activity of branched P450cam in reversed micelles showed a linear relationship with protein concentration (Figure 2a). The initial activity was strongly dependent on water content (W_0_ = [water]/[surfactant]), which determines the radius of the water pool. The optimum W_0_ was 16.7 (Figure 2b). The water pool at a W_0_ value of less than 16.7 would be smaller than the space required for efficient intramolecular electron transfer and/or catalytically active conformations of the fusion protein. The decrease of initial activity with an increase of W_0_ may be caused by the decrease of *d*-camphor concentration in the water pool due to its superior solubility in isooctane and its poor solubility in water. In the reversed micellar system, the *K*_m_ value for *d*-camphor was 1.3 mM (Figure 3), which is a thousand times larger than that in aqueous solution [17], and the initial activity strongly depended on the concentration of *d*-camphor at concentrations under 5 mM. This indicates that the concentration of *d*-camphor in the water pool was very low and that the initial activity was sensitive to a decrease of *d*-camphor concentration with the increase of water content in a reaction mixture containing 5 mM *d*-camphor. The UV-Vis spectrum of the branched P450cam showed a shoulder peak at around 420 nm (Figure 4), which indicates the *d*-camphor unbound state of P450cam. These results strongly supported the speculation that the concentration of *d*-camphor in the water pool, where branched P450cam was located, was low.

The NADH consumption activities of equimolar mixtures of PdR, PdX and P450cam were negligible compared with branched P450cam at any W_0_ value (Figure 2b). Reversed micelles can be regarded as nano-reactors, and the coexistence of all component proteins in one micelle enables extremely high local concentrations of proteins and efficient electron transfer to P450cam. However, the negligible NADH consumption indicates that the co-localization probability of the three proteins, PdR, PdX and P450cam, in one micelle is quite low unless an extremely concentrated protein mixture is injected into the surfactant solution in the organic solvent. Therefore, the branched fusion protein composed of P450 and its electron transfer proteins is effective for use in reversed micellar systems. 

### 2.2. NAD^+^ reduction by A. pernix ADH in reversed micelles

Cofactor regeneration is a major challenge to practical applications of cofactor-dependent enzymes because it is too expensive to use stoichiometric amounts of cofactors. To overcome this problem, several cofactor regeneration methods have been developed [21,22,23,24,25,26]. In enzymatic NAD(P)H-regeneration systems, various dehydrogenases, such as alcohol dehydrogenase [27,28,29], glucose-6-phosphate dehydrogenase [30], glycerol dehydrogenase [31], formate dehydrogenase [32] and phosphite dehydrogenase [33,34] have been used. However, dehydrogenases are generally unstable against heat and organic solvents. We have previously reported that an NAD(H)-dependent alcohol dehydrogenase from a hyperthermophilic archeon *Aeropyrum pernix* (*A*. *pernix* ADH) is highly thermostable [35]. Interestingly, *A*. *pernix* ADH prefers aliphatic long chains, such as hydrophobic alcohols and is activated by hydrophobic organic solvents [36]. Therefore, the enzyme is appropriate for NADH-regeneration in media containing hydrophobic organic solvents.

**Figure 5:**
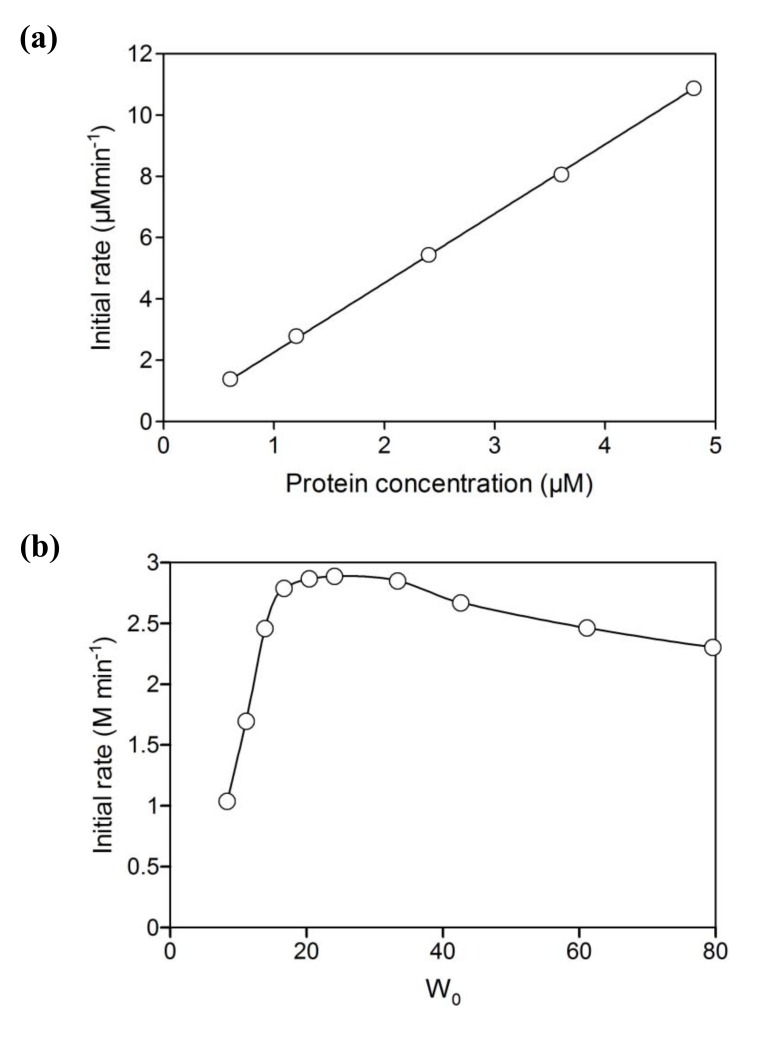
(a) Relationship between the initial activity and the concentration of *A*. *pernix* ADH with 70 μM NAD^+^ at a W_0_ of 16.7. (b) Effect of W_0_ on the initial activity of *A*. *pernix* ADH at 1.2 μM.

*A*. *pernix* ADH showed NAD^+^ reduction activity in a CTAB/2-pentanol/isooctane reversed micellar system without the addition of other alcohols, and had a linear relationship with the concentration of the protein (Figure 5a). This indicated that 2-pentanol was dissolved in the water pool and used as a substrate by *A*. *pernix* ADH, as well as being localized at the interface of micelles as a co-surfactant. 

ADH activity was dramatically decreased at low W_0_ values, similar to that of branched P450cam (Figure 5b). To have active conformations in a reversed micellar system, sufficient space in the water pool is required to accommodate the size of the enzyme. In contrast to branched P450cam, the activity of *A. pernix* ADH showed a broad optimum peak against W_0_ and was not significantly decreased at high W_0_. This difference was caused by the difference in solubility between 2-pentanol and *d*-camphor. Comparatively higher solubility of 2-pentanol in water might be sufficient to supply higher concentrations of the substrate. 

**Figure 6:**
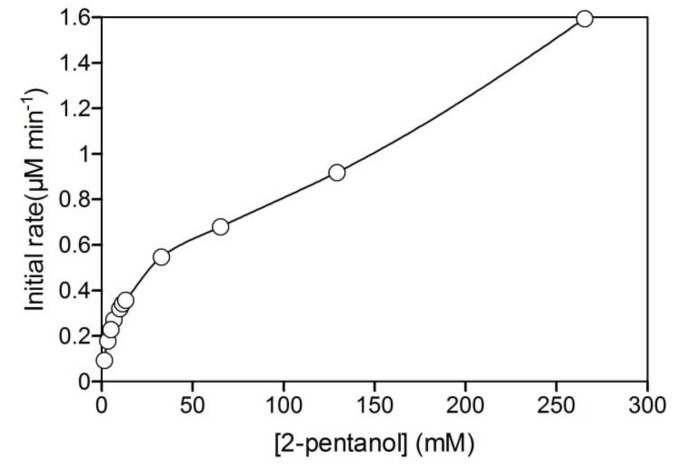
Relationship between the initial reaction rate of *A*. *pernix* ADH and the concentration of 2-pentanol in 50 mM potassium phosphate buffer, pH 7.4, containing 150 mM KCl. The reaction mixture contained 1.2 μM *A*. *pernix* ADH and 70 μM NAD^+^.

The maximum activity of *A*. *pernix* ADH in the reversed micellar system was much higher than that in aqueous solution. There are two possible reasons for this; i) activation by the hydrophobic environment and ii) substrate activation by 2-pentanol. We have previously shown that *A*. *pernix* ADH has high catalytic activity in a hydrophobic environment by the addition of water-immiscible organic solvents [36]. The water pool in the reversed micelles should be hydrophobic due to the presence of isooctane and this water pool environment in the reversed micelles may activate the enzyme. *A*. *pernix* ADH also showed substrate inhibition by primary alcohols and substrate activation by secondary alcohols (unpublished data), properties that have also been reported for horse liver ADH [37,38]. Nonetheless, substrate activation was also observed when using 2-pentanol as a substrate in water (Figure 6), although the activity of *A. pernix* ADH in the reversed micellar system was higher than that in water in the presence of 250 mM of 2-pentanol. 

### 2.3. Coupled reaction by branched P450cam and A. pernix ADH

**Figure 7:**
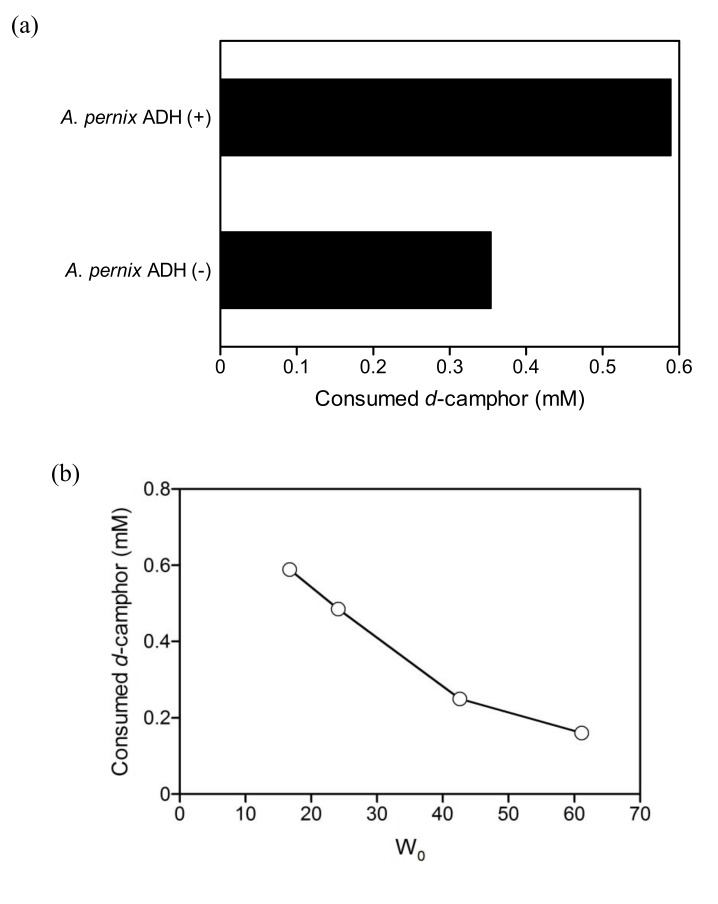

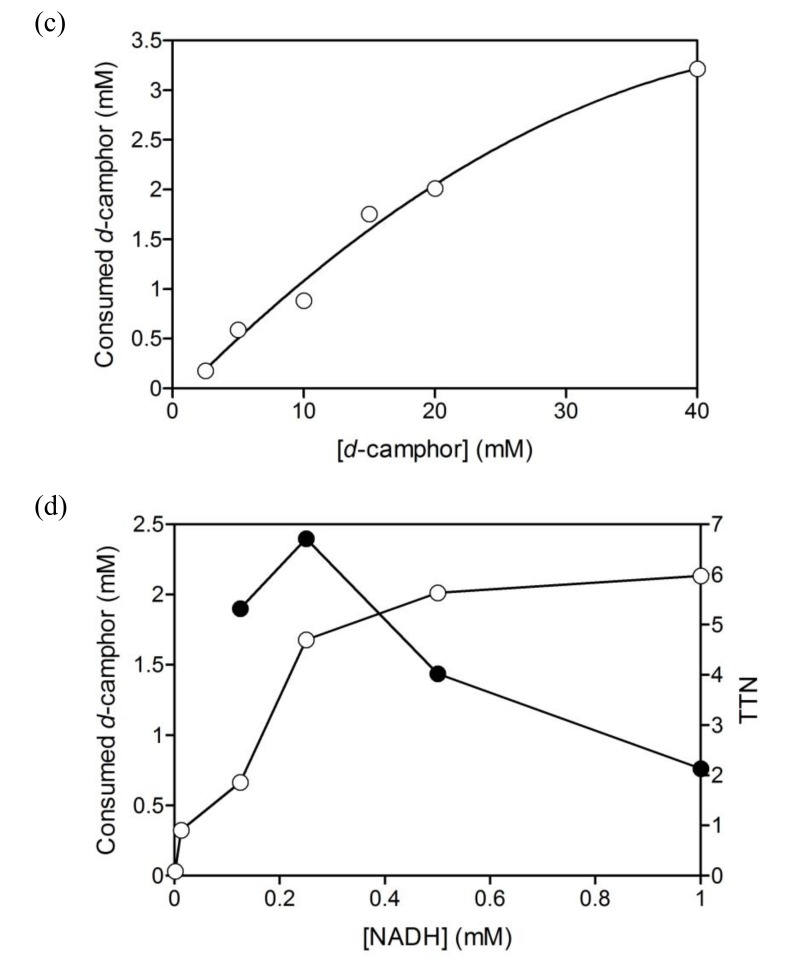
(a) The consumption of *d*-camphor by branched P450cam (1 μM) in the reversed micellar system after 24 h in the presence (37 μM) and absence of *A*. *pernix* ADH with 1 mM NADH and at a W_0_ of 16.7. (b) Effect of W_0_ on the consumption of *d*-camphor after 24 h in the coupled reaction containing 1 μM branched P450cam and 37 μM *A. pernix* ADH with 1 mM NADH. (c) Effect of *d*-camphor concentration on the consumption of *d*-camphor after 24 h in the coupled reaction containing 1 mM NADH and at a W_0_ of 16.7. (d) Effect of initial NADH concentration on the consumption of *d*-camphor (open circles) and on the TTN of NADH (closed circles) after 24 h in the coupled reaction containing 20 mM *d*-camphor and at a W_0_ of 16.7.

*A*. *pernix* ADH promoted the hydroxylation of *d*-camphor by branched P450cam (Figure 7a). The consumption of *d*-camphor by the coupled reaction (Figure 7b) and the initial rate of *d*-camphor consumption by branched P450cam (Figure 2b) showed similar W_0_-dependency. In addition to these results, the consumption of *d*-camphor increased with the increase in substrate concentration and the total turnover number (TTN) of NADH exceeded 1.0 when *d*-camphor was greater than 10 mM (Figure 7c). These results clearly indicate that NADH was regenerated by *A*. *pernix* ADH and suggest that the rate limiting step in the coupled reaction was hydroxylation by branched P450cam. 

The regeneration of NADH by *A*. *pernix* ADH could allow a reduction in the initial amount of NADH in the reversed micellar system and the TTN reached a maximum at an NADH concentration of 0.25 mM (Figure 7d). The consumption of *d*-camphor dramatically decreased when the initial NADH concentration was less than 0.25 mM. This indicates a decrease in the hydroxylation rate of *d*-camphor by branched P450 at NADH concentrations of less than 0.25 mM, although the *K*_m_ value for NADH with respect to branched P450cam is 2.5 μM in aqueous solution [17] and the concentration in the water pool must be higher than 0.25 mM. NAD^+^, which is a product of NADH oxidation, is known to be a competitive inhibitor of PdR. In contrast, NAD^+^ reduction catalyzed by *A*. *pernix* ADH is not thought to be strongly inhibited by NADH. Substrate activation by *A*. *pernix* ADH, shown in Figure 6, indicates that the release of NADH from the enzyme-NADH complex is a rate limiting step in the secondary alcohol oxidation reaction and that the release is accelerated via the enzyme-NADH-alcohol complex in the presence of secondary alcohols [38]. Therefore, the NAD^+^ inhibition of branched P450cam might be relatively strong at low concentrations of NADH and an increase of NADH concentration at steady state by an increase of initial NADH concentration might recover the reaction rate.

**Figure 8:**
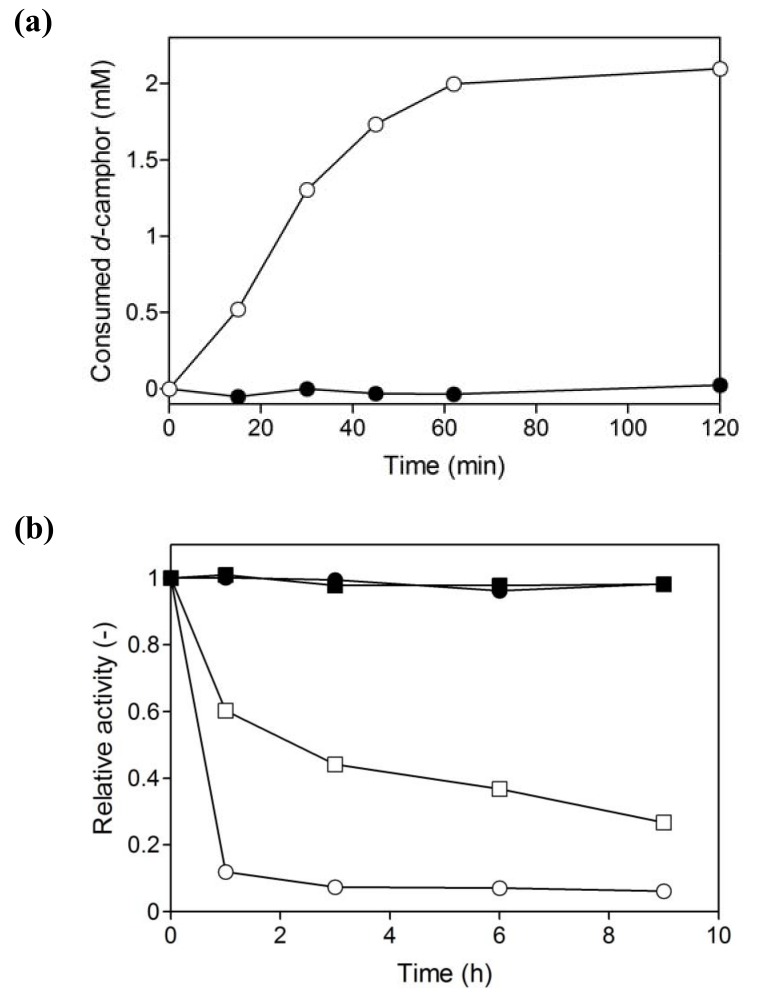
(a) Time course of *d*-camphor consumption by branched P450cam (open circles) and by an equimolar mixture of P450cam, PdX and PdR (closed circles) in the presence of *A*. *pernix* ADH at a W_0_ of 16.7. (b) The stability of branched P450cam (open symbols) and of *A*. *pernix* ADH (closed symbols) in the reversed micellar system at a W_0_ of 16.7 (circles) and in an aqueous solution (squares).

Within 1 h, the coupled reaction was stopped although the hydroxylation of *d*-camphor was not complete and the reaction mixture still contained abundant substrate (Figure 8a). Branched P450cam in the reversed micellar system was more unstable than in an aqueous solution (Figure 8b). The residual activity of branched P450cam in the reversed micellar system was 10% after 1 hour, while it was more than 60% in the aqueous solution. 

Binding of *d*-camphor stabilizes P450cam [39]; therefore, the low concentration of *d*-camphor in the water pool may be one reason for the instability. In contrast, *A*. *pernix* ADH was quite stable in the reversed micellar system as well as in the aqueous solution. The enzyme retained complete activity both in the reversed micellar system and in water for at least 2 weeks (date not shown). Therefore, the incomplete hydroxylation reaction in the reversed micellar system is most likely due to inactivation of branched P450cam.

## 3. Experimental Section

### 3.1. Protein preparations

Branched P450cam was prepared as described previously [17]. An alcohol dehydrogenase from *A*. *pernix* was expressed as described previously [35] and purified as follows. Cells were resuspended in 10 mM potassium phosphate buffer (pH 7.2) containing 0.1 mM 4-(2-aminoethyl)benzenesulfonyl fluoride (AEBSF) and disrupted with ultrasonication. The cell debris was removed by centrifugation at 22,000 g for 30 min. The supernatant was incubated at 75 ºC for 15 min. Aggregated proteins were removed by centrifugation at 22,000 g for 30 min. The supernatant was subjected to anion exchange chromatography on a DEAE Sepharose CL 6B column. The protein was eluted with a linear gradient of KCl (0–500 mM). The fractions containing the protein were collected and concentrated by ultrafiltration using Amicon Ultra filter units (100 kDa NMWL). The concentrated protein was subjected to gel-filtration chromatography on a Superdex 200 HR 10/30 column pre-equilibrated with 50 mM potassium phosphate buffer (pH 7.4) containing 150 mM KCl. The purified protein was concentrated with Amicon Ultra filter units (100 kDa NMWL).

### 3.2. Preparation of reversed micelle solutions

A reversed micelle starter solution was prepared by vigorous shaking of a suspension containing 0.3 M cetyltrimethylammonium bromide (CTAB), 1.8 M 2-pentanol and 50 mM potassium phosphate buffer (pH 7.4) with 150 mM KCl until a clear solution was obtained. This starter solution contained 1.7 mM water molecules. Working reversed micelle solutions were prepared by adding enzyme solution, cofactor solution, buffer, isooctane, and/or *d*-camphor solution in isooctane.

### 3.3. Activity assays

Activities of branched P450cam were estimated from NADH consumption rates, which were determined by initial absorption changes at 340 nm after adding a working reversed micelle solution containing proteins and *d*-camphor to a working reversed micelle solution containing NADH. Activities of *A*. *pernix* ADH were estimated from NADH generation rates after adding a working reversed micelle solution containing *A*. *pernix* ADH to a working solution containing NAD^+^. All experiments were performed at 25 ºC. 

### 3.4. Hydroxylation of d-camphor with cofactor regeneration

Reactions were initiated by adding buffer solution containing enzymes to a working reversed micelle solution containing NADH and *d*-camphor to a total volume of 750 μL. After adding bromocamphor solution in isooctane as an internal standard, 600 μL of 8.3% trifluoroacetic acid solution was immediately added with vigorous shaking to stop reactions. The water layer was washed with 200 μL of CH_2_Cl_2_ three times. The CH_2_Cl_2_ washes and the organic layer were combined and dried by adding anhydrous magnesium sulfate. After removing magnesium sulfate with a 0.22 μm filter, the sample was concentrated under a flow of N_2_ gas. Consumed *d*-camphor was estimated from the remaining *d*-camphor, which was determined by gas chromatography equipped with a flame ionized detector (HP 6850, Agilent, USA). An aliquot (approximately 1 μL) was applied to a CHIRALDEX G-TA column (25 m × 0.25 mm I.D.; Advanced Separation Technologies, Whippany, NJ, USA).

## 4. Conclusions

We have clearly shown the potential of site-specific enzymatic cross-linking in the utilization of multi component enzymes in a reversed micellar system. Branched P450cam is a fusion protein with a branched structure consisting of P450cam, PdR and PdX that showed catalytic activity in CTAB/2-pentanol/isooctane due to its self-sufficiency. In contrast, P450cam did not show catalytic activity in the reversed micellar system containing its electron transfer proteins, PdR and PdX because each protein is likely to exist in separate micelles. Although reversed micellar systems can increase the concentrations of hydrophobic substrates, the low concentration of substrates in the water pool might dominate the reaction rate in large micelles. *A*. *pernix* ADH was highly thermostable in the reversed micellar system and was also activated. This property suggests that this alcohol dehydrogenase is suitable for NADH-regeneration in reversed micellar systems. Indeed, the consumption of *d*-camphor by branched P450cam was promoted in the presence of *A*. *pernix* ADH, and the NADH oxidized by branched P450cam could be successfully regenerated.
